# Significance of PSCA as a novel prognostic marker and therapeutic target for cancer

**DOI:** 10.1186/s12935-024-03320-6

**Published:** 2024-04-16

**Authors:** Tina Nayerpour Dizaj, Abolfazl Doustmihan, Behnaz Sadeghzadeh Oskouei, Morteza Akbari, Mehdi Jaymand, MirAhmad Mazloomi, Rana Jahanban-Esfahlan

**Affiliations:** 1 Department of Medical Biotechnology, Faculty of Advanced Medical Sciences, Tabriz University of Medical Sciences, Tabriz, Iran; 2 Department of Reproductive Biology, Faculty of Advanced Medical Sciences, Tabriz University of Medical Sciences, Tabriz, Iran; 3 Nano Drug Delivery Research Center, Health Technology Institute, Kermanshah University of Medical Sciences, Kermanshah, Iran; 4 Student Research Committee, Kermanshah University of Medical Sciences, Kermanshah, Iran

**Keywords:** PSCA expression, Polymorphisms, Prognostic biomarker, Cancer therapy

## Abstract

One of the contributing factors in the diagnosis and treatment of most cancers is the identification of their surface antigens. Cancer tissues or cells have their specific antigens. Some antigens that are present in many cancers elicit different functions. One of these antigens is the prostate stem cell antigen (PSCA) antigen, which was first identified in the prostate. PSCA is a cell surface protein that has different functions in different tissues. It can play an inhibitory role in cell proliferation as well as a tumor-inducing role. PSCA has several genetic variants involved in cancer susceptibility in some tissues, so identifying the characteristics of this antigen and its relationship with clinical features can provide more information on diagnosis and treatment of patients with cancers. Most studies on the PSCA have focused on prostate cancer. While it is also expressed in other cancers, little attention has been paid to its role as a valuable diagnostic, prognostic, and therapeutic tool in other cancers. PSCA has several genetic variants that seem to play a significant role in cancer susceptibility in some tissues, so identifying the characteristics of this antigen and its relationship and variants with clinical features can be beneficial in concomitant cancer therapy and diagnosis, as theranostic tools. In this study, we will review the alteration of the PSCA expression and its polymorphisms and evaluate its clinical and theranostics significance in various cancers.

## Introduction

The PSCA (Prostate stem cell antigen) antigen, also known as PRO232, is a small glycoprotein antigen that binds to the cell surface via the GPI interface (glycosylphosphatidylinositol) and belongs to the Thy-1/Ly-6 family presenting 30% homology with stem cell antigen -2 (SCA-2). Members of the Thy-1/Ly-6 family bind to the cell surface by GPI [1]. In 1998, using a lapc-4 prostate cancer xenograft model PSCA gene was first discovered and located on chromosome 8q24.2 [2]. Overlapping of two human PSCA clones and phage λ by fluorescence in situ hybridization analysis revealed a specific and unique labeling on chromosome 8, in that, 97% of the signals were related to chromosome 8q24 with 87% specificity in the region of 8q24.2 [1]. Moreover, Sothern blot analysis of LAPC-4 genomic DNA has shown that PSCA is encoded by a single-copy gene and contains 3 exons and 2 introns. PSCA is located on chromosome 8 q24.2, 15 MB away from the C-Myc oncogene [3].

The PSCA gene encodes a 123 amino acid protein with a molecular weight of 10–24 kDa that has a signal sequence in the N-terminal region and a GPI-binding sequence in the C-terminal region, as well as multiple N glycosylation sites [1]. To confirm the binding of PSCA via the GPI anchor to the cell surface, [1] cells were treated with GPI-specific phospholipase C (PLC). The affinity-purified polyclonal antibody was also applied against a unique PSCA peptide, which lacked a glycosylation site, to prove the presence of N-glycosylation sites. Some GPI-anchored proteins have two secretory and membrane-bound forms. PSCA secretory form has lower molecular weight due to the lack of disulfide bonds related to GPI [1, 4, 5].

PSCA protein expression is influenced by a variety of factors, including PSCA gene amplification [6], transcriptional control [7], intracellular signal transduction [8], and PSCA expression possibly by cell-cell contact and several pathways such as protein kinase C [9]. One study showed that the YY1 multifunctional transcription factor binds directly to the PSCA promoter and regulates its expression in androgen-dependent cell lines [10]. Another regulatory mechanism is androgen, which is likely to regulate PSCA expression, and the rationale for this claim is the presence of androgen-responsive elements in the promoter region of the PSCA gene [11]. The next mechanism is telomerase regulation, as the down-regulation of PSCA in telomerase-induced urothelial cells is verified [12].

PSCA mRNA expression in normal prostate tissue is more common in basal cells and conflicting reports of non-expression or low expression in secretory cells [1]. PSCA mRNA is also found at low to very low levels in the placenta, kidneys, and small intestine, respectively [13]. PSCA protein expression is expressed in a small number of normal tissues, including prostate epithelial tissue (in both basal and secretory layers), urethral epithelium (restricted to umbilical cells), and neuroendocrine cells of gastric and colon (confirmed by double staining with chromogenin), weakly in renal ducts except glomerular layer, placental trophoblast, and inconsistent reports of its expression in normal pancreatic tissue [13–15]. The high expression level of PSCA is observed in several cancers, including non-small cell lung carcinoma (NSCLC), clear cell renal cell carcinoma (CC-RCC), and other cancers.

In this study, we will highlight PSCA significance as a novel prognostic and therapeutic cancer biomarker. We review the alteration of the PSCA expression and its polymorphisms in different cancers and evaluate its clinical and theranostics significance in various cancers.

## PSCA function, expression and mechanism of action

PSCA is a protein called Jekyll and Hyde because PSCA has different functions in different human tissues, which means that PSCA can have functional variability depending on the type of tissue, cell status, normal or malignant [16]. As this protein has both an up-regulated and a down-regulated pattern in some tissues, it can have both tumorigenic and tumor-suppressive roles [16]. Expression levels of PSCS in normal, as well as different cancerous tissues, are provided in Table 1, and Table 2 respectively.

**Table 1:** mRNA and protein expression pattern of PSCA in various normal tissues

Tumor type	Cell type	Gene/protein	Reference
Prostate	Basal cells	mRNA	[1]
	Secretory cells	mRNA	
Kidney	Kidney	mRNA	[45]
	Tubules	Protein	
Bladder	Superficial umbrella cell	mRNA	[92]
	Superficial layer of transitional epithelium	Protein	
Gastric	Mucosa and isthmus of gastric glands	mRNA	[13] [45–63]
	Neuroendocrine cells	Protein	
Colon	Neuroendocrine cells	Protein	[111]
Pancreas	Islets of the pancreas (α,β,δ and pp cells)	Protein	[95]
Brain	Neural and plexus and cortex	Protein	[80–84]
gallbladder	Epithelium of gallbladder	Protein	[96]
Oral squamous cells	Keratin epithelial cells	mRNA	[90]
Esophagus	Epithelial cells of the esophagus	Protein	[95]

**Table 2:** Up regulation and down-regulation of PSCA expression in different cancer tissues

Tissue	Type of cancer	expression	Reference
Breast	invasive micropapillary carcinoma (IMPC) and invasive ductal carcinoma of nonspecial type (IDC-NST) Invasive lobular carcinoma (ILC)	Up-regulated down-regulated	[55]
Cervical	Cervical adeno carcinoma (CAC)	Up-regulated	[94]
Endometrial	Endometrial adenocarcinoma (EAC)	Up-regulated	[91]
Gestational	Gestational trophoblastic neoplasia (GTN)	Up-regulated	[64]
Lung	Non-small cell lung carcinoma (NSCLC)	Up-regulated	[85]
Renal cell	Clear cell renal cell carcinoma (CC-RCC)	Up-regulated	[116]
Brain	Brain	Up-regulated	[81]
ovarian	Ovarian mucinous carcinoma	Up-regulated	[21]
Bladder	Bladder cancer	Up-regulated	[92]
Nasopharyngeal	Nasopharyngeal carcinoma (NPC)	Down-regulated	[86]
Pancreas	Pancreatic cancer	Up-regulated	[78]
Gallbladder	Gallbladder carcinoma	Down-regulated	[96]
Esophagus	Esophagus carcinoma	Down-regulated	[104]
Gastric	Gastric cancer	Down-regulated	[64]

As mentioned earlier, PSCA belongs to the Thy-1/Ly-6 family [1]. Proteins in the Thy-1 family are involved in T cell activation [17], as well as the proliferation and survival of stem cells and the response to cytokines and growth factors [18]. Members of the Ly-6 family are involved in carcinogenesis [19, 20], cell activation [21] and cancer cell adhesion [22]. PSCA is also involved in cellular signaling, cell proliferation, cell cycle regulation, immune response, and disturbing the balance of pro-inflammatory and anti-inflammatory signals [16].

**Fig. 1:**
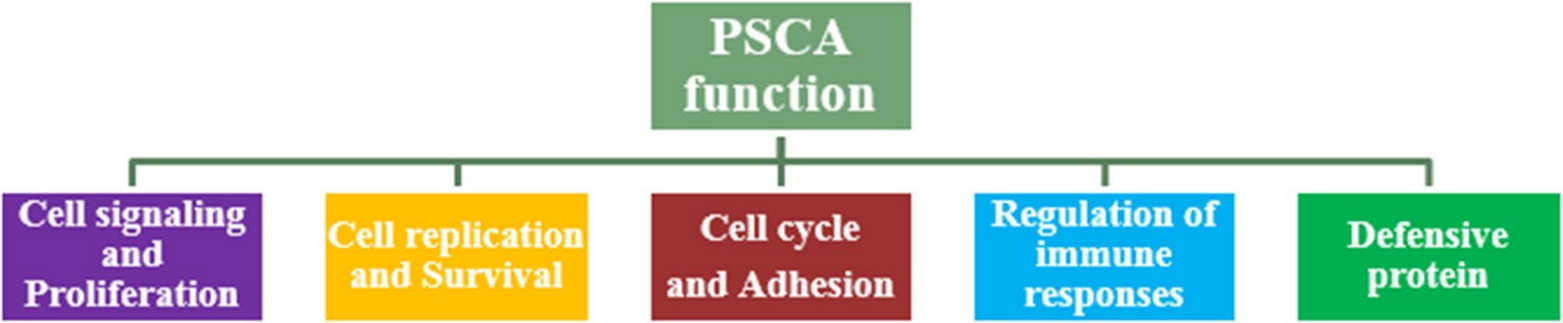
Schematic view of PSCA function

Evidence for the role of PSCA in the immune system shows that inoculation of TRAMP mice with a PSCA-based vaccine provides long-term protection against cancer spread without raising autoimmune reactions [23]. Also, PSCA or PSCA-expression plasmid injection into mice containing transplant-prostate tumor cells, inhibits tumor growth by inducing CD8 + T-cell immune responses [24, 25]. Another possibility is that PSCA may affect pro-inflammatory and anti-inflammatory signals by interfering with nAchRs7α [26]. Also, PSCA may interact with IFNα / β and act as a defense protein [27] (Fig. 1).

Given its structure, several mechanisms have been proposed for PSCA function. For one, PSCA may form complexes with other proteins, which have intracellular domains and transmembrane proteins that need to activate their downstream targets. PSCA has an activating type I and II extracellular receptor, which binds to TGFβ and is responsible for key functions [28]. Another possible mechanism is through phospholipase C, wherein by breaking the GPI anchor, PSCA can be released from the cell surface and in the secretory form, PSCA function through the receptor-dependent signaling pathway [16] 

## Types of PSCA polymorphisms

Several copies of the gene locus in a population are called polymorphisms. Gene polymorphisms can occur in any region of the gene. Types of these single nucleotide polymorphisms (SNPs) can be found at the National Center for Biotechnology Information (NCBI) and SNP databases (http://www.ncbi.nlm.Nih.gov/projects/SNP/). Most polymorphisms are silent, meaning that they do not alter the function or expression of the gene, but some are visible. Numerous studies have shown that screening and identifying SNPs as biomarkers can play a role in genetic modification. There are 464 recorded SNPs for PSCA gene, which is involved in cell adhesion, proliferation, and survival [2]. In the study conducted by Cui, H et al. [29], out of 29 genetic variants reported in the PSCA gene, 22 variants are associated with more than one cancer. Specially rs2294008, which is related to 9 cancers and four non-neoplastic diseases. The remaining 7 variants are related to one type of cancer. In this study, 6 PSCA genetic variants related to the risk of cancer and non-neoplastic disease were investigated. This observation can be explained by clonal heterogeneity of PSCA which shows different level of none, moderate and high heterogeneity in cancer (Table 3).

**Table 3:** Functional annotation of the six significant variants in the PSCA gene on chromosome 8q24

Variant	Annotation	Position	Clonal heterogeneity		
			None	Moderate	High
rs2294008	Missense	143761931	Duodenal ulcer and gastric ulcer	–	Gastric cancer
			–	Gastritis	–
rs138377917	Stop-gain	143763531	–	–	Gastric cancer
rs2976392	Intergenic	143762932	–	–	Gastric cancer
rs9297976	Intergenic	143752235	Gatric cancer	–	–
rs2976391	Intergenic	143762724	–	Gastric cancer	–
rs12155758	Intergenic	143765885	Bladder cancer	–	–

One of the most common PSCA SNPs is rs2294008, which is located in exon 1 of the PSCA gene and is a missense variant that changes the PSCA start codon. Rs2294008 has two alleles: The T allele encodes the long PSCA and through an additional fragment of 9 amino acids in the N-terminal region changes the localization of PSCA from the cytoplasm to the cell surface. The C allele encodes the short PSCA which is localized in the cytoplasm [30].

Genetic variants in PSCA play an important role in PSCA function and can alter the biological function of this protein by altering subcellular localization and stability [31]. It appears to be an association between PSCA variants and some cancers, as studies in Japanese and Korean populations have shown that T < C rs2294008 and G > A rs2976392 are associated with an increased risk of bladder and gastric cancer [32]. Also, the results of a meta-analysis study showed that the increased risk of cancer with rs2294008 C > T polymorphism level is observed especially in patients with gastric and bladder cancer, while rs2976392 G > A polymorphism is observed in patients with gastric cancer. Also, their statistical analysis showed that these two SNPs are more significantly associated with stomach and bladder cancer than other cancers. It was stated that different types of cancers have different aspects of features that lead to different statistical results and the location of a particular polymorphism is probably associated with a particular type of tumor. Subgroup analysis based on ethnicity also showed that rs2294008 polymorphism C > T has the highest risk of cancer in the Asian and Caucasian populations, while rs2976392G > A only affects the Asian population, and their rationale for this is that different nationalities have diverse genetic backgrounds that may have a different SNPs for cancer development [33]. Also, a meta-analysis of PSCA variants was conducted to test whether there is a strong relationship between the linkage disequilibrium (LD) factor and several different variants for the occurrence of one type of cancer in several different types of populations. Results showed that functional mechanisms that associate these variants with the increased risk of the same type of cancer in these populations are similar. The mechanism of action works differently and can be a justification as to whether some variants in some nationalities are involved in the development of some type of cancer and have no role in other nationalities Table 4 [29].

**Table 4:** Associations between variants and risk of cancer and non-neoplastic disease

Variant	Cancer type	Non-neoplastic disease
rs2294008 T allel	Increased risk of gastric cancer, non-cardia gastric cancer, diffuse-type gastric cancer, bladder cancer and reduced risk of esophageal cancer	Increased risk of gastritis, reduced risk of duodenal ulcer
rs138377917	Gastric cancer	–
rs2976392	Gastric cancer	–
rs9297976	Gastric cancer	–
rs2976391	Gastric cancer	–

## PSCA expression and polymorphisms in different cancers

In this section, we aim to review the significance of PSCA in different types of tumors. Relevant clinical studies are also provided in Table 5**.**

**Table 5:** PSCA and its function in different types of cancer

Cancer type	Sample size	Type of analysis	Key results	Ref
Breast	Patients n = 405	IHC	There is no correlation between PSCA protein expression and estrogen, or progesterone receptor status, but there is a strong correlation between this protein expression and Her2/neu receptor status. Also, there is no association between PSCA-protein expression and PFS or OS	[54]
Gastric	Patients and their MNGT n = 438 pairs	Western blot, IHC	PSCA acts as a tumor suppressor and has a low expression level in the GC. Low PSCA expression in GC is associated with poor prognosis and OS	[55]
Gastric	well and moderately differentiated n = 37 poorly differentiated and undifferentiated n = 63	RT qPCR	The expression of PSCA was increased in moderately and well-differentiated tumors, whereas it was reduced in poorly differentiated and undifferentiated tumors and there was no significant correlation between PSCA expression and clinicopathological parameters, such as age, gender, tumor size, TNM stage, invasion, and lymph node metastasis	[56]
Pancreas	Patients n = 94	IHC, western blot	PSCA is highly expressed in PDAC, and high PSCA expression is significantly correlated with tumor size and nodal metastasis, OS and PFS	[57]
Pancreas	patients n = 40 controls n = 60	Flowcytometry	PSCA is up–regulated and 80% of subjects with pancreatic cancer show significantly elevated levels of IgGs against at least one of the three peptides of PSCA at positions 2–11, 85–95, and 109–118	[58]
Non-small cell lung cancer	Primary tumors n = 97 Metastatic lymph nodes n = 21	IHC, SiRNA against PSCA was applied to reduce its expression	PSCA is highly expressed in 97% of primary tumors and 100% of metastatic lymph nodes and their intensity is correlated with tumor stage. PSCA overexpression is negatively correlated with 5 year DFS.and DFS curves of patients with adenocarcinoma according to the PSCA expression	[59]
Nasopharyngeal carcinoma	Human nasopharyngeal carcinoma cell lines (S26, S18, 6–10B, and 5–8 F)	Knockdown and overexpression of PSCA	PSCA is a key player in NPC metastasis by serving as a downstream target of Slug to participate in the EMT. Patients with high PSCA expression in their primary tumors had better OS and DMFS rates compared with those with low PSCA expression	[60]
Endometrial adenocarcinoma	64 (EAC) and paired normal endometrium	IHC	PSCA can be a marker for screening patients over 54 years of age due to its age-related relevance and association with tumor grade and lymph node metastasis. PSCA is involved in the development and progression of EAC cancer and helps diagnose tumor malignancy, while TBX2 has a key role in PSCA up-regulation	[61]
Esophagus	300 pairs of primary ESCC tissue samples and their corresponding non-tumorous tissues	IHC	PSCA is frequently downregulated in ESCC and acts as a tumor suppressor by stabilizing and facilitating nuclear translocation of (RB1CC1). Its downregulation correlates with poor survival outcomes in ESCC	[62]
Colorectal	Patients n = 388 controls n = 496	IHC and genotyping	PSCA has no role in the initiation or progression of colorectal carcinogenesis	[63]
Clear cell renal cell carcinoma	Patients n = 81 controls n = 73	RT-PCR, IHC	PSCA is associated with carcinogenesis and progression of CC-RCC. A high PSCA expression level is predictive of poor survival in patients	[64]
Transitional cell carcinoma	Patients n = 142 controls n = 32	IHC	PSCA is expressed by a majority of superficial and muscle-invasive transitional cell tumors, human TCCs, particularly CIS and superficial tumors	[65]
Gestational trophoblastic	First-trimester placentas n = 10 HM that subsequently regressed n = 36 HM that developed GTN n = 11	RT-PCR, IHC	Overexpression of PSCA is associated with the development of GTN in Hydatidiform mole (HM)	[66]

### PSCA and prostate cancer

Prostate cancer is one of the most common cancers in the world. Prostate cancer begins when prostate gland cells grow uncontrollably [34, 35]. Due to the high prevalence of this type of cancer, it is necessary to seek solutions for early diagnosis and treatment. This can be achieved by recognition of specific biomarkers to identify this type of cancer. Several markers have been identified for the prostate, including PSA, which performs poorly in the diagnosis of mild to advanced prostate cancer, prostate-specific membrane antigen (PSMA) is used to identify metastatic cells and is intended as a target for mAb and other immunological therapies. Her2/neu is one of the growth factor receptors associated with prostate cancer, which can be androgen-independent and as an important signaling molecule is overexpressed in 80% of metastatic prostate cancer [36–39]. Other receptors such as C-met and Urokinase plasminogen activator receptors may act as modulators of prostate cancer metastases [40–42]. Finally, the most important marker that appears to have a potential role in the development of prostate cancer is PSCA. As mentioned earlier, PSCA antigen is a cell surface antigen that can be effective for many immunological strategies [43].

The expression of PSCA protein and mRNA in normal and cancerous prostate tissue has been investigated by the in-situ hybridization (ISH) method using antisense RNA. Lack of PSCA mRNA expression is seen in stroma and secretory cells [1] and its expression in basal cells suggests that PSCA may be a marker of prostate specific stem/progenitor cells [44]. Basal cell epithelium contains progenitor cells that are required for the final differentiation of secretory cells. Using the cytokeratin marker, it was determined that there are at least two types of cellular subpopulations in the basal cell epithelium and PSCA is secreted by only one subset of basal cells [45].

Using monoclonal antibodies (mAbs), PSCA protein expression is observed in secretory cells and endocrine cells in addition to basal cells. To explain this difference, two possibilities have been suggested [46]. PSCA mRNA is transcribed in basal progenitor saliva and that the PSCA protein resides in these cells and differentiates into secretory cells. The next possibility is that the PSCA protein is post-transnationally transported from basal cells to secretory cells. ISH analysis has shown that PSCA mRNA is expressed in more than 80% of prostate cancers and this expression is much higher than the adjacent normal glands. Likewise, mAbs confirmed 80% overexpression of PSCA and its association with Gleason score, pathologic stage and androgen-independence [46].

Because PSCA is an androgen-responsive gene, the expression of PSCA in the prostate is modulated by interaction with the androgen receptor (AR) via an androgen-responsive element (ARE) [11]. On the other hand, the YY1 multifunctional transcription factor binds directly to the PSCA promoter and regulates its expression in androgen-dependent cell lines [10]. There are two binding sites for YY1 upstream of the PSCA gene, where binding to the upstream region suppresses PSCA promoter activity, while downstream binding stimulates PSCA gene promoter activity [10]. Analysis of ISH and immunohistochemistry (IHC) showed that the expression of PSCA protein and mRNA in benign prostatic hyperplasia (BPH) and low-grade prostatic intraepithelial neoplasia (PIN) is weak or not expressed, but has a very high expression in high-grade form (HGPIN) [46]. Plus, increased protein and mRNA expression of PSCA is associated with tumor grade (poor differentiation), stage and progression to androgen-independence [6].

Although the exact mechanism of action of PSCA in prostate cancer is not fully understood, two theories have been proposed. One possibility is amplification of the PSCA gene [6]. PSCA is located near the C-myc oncogene, which is amplified in more than 20% of metastatic and recurrent prostate cancers [47, 48]. PSCA gene and copy numbers of C-myc gene coamplified in 25% of tumors [49], which shows the association between PSCA overexpression with PSCA and C-myc coamplification. Another possibility is PSCA mRNA overexpression [6]. PSCA antigen is likely to be involved in tumorigenesis and clinical progression of prostate cancer, by affecting cell proliferation and transformation [6]. PSCA also potentiates prostate cancer by increasing C-myc expression through the PI3K/AKT signaling pathway [50].

### PSCA SNPs in prostate cancer

In addition to the role of PSCA in prostate cancer, PSCA variants also appear to play a role in the development of this cancer. The study of three genetic variants rs1045531, rs3736001 and rs2294008 in the incidence of PCa and their relationship with factors such as age, ethnicity and clinical parameters such as PSA, Gleason score, and pathologic stage has shown different manifestations. Genotype AA rs1045531 has a higher risk of susceptibility than genotype CC in the population of South Korea, and this variant is associated with PSA in this population and has no association with the Gleason degree [51]. Meanwhile, analysis of rs1045531 variant in the Chinese population undergoing biopsy shows that the heterozygous AC model and the predominant AA/AC model play a major role in the development of PCa, and the homozygous and recessive model has no association with prostate cancer in Chinese population, and this variant is also associated with PSA, and Gleason degree in the Chinese population undergoing biopsy [52]. Other studies show that people who carry the CCCAGGTACGG and CGA haplotypes have a higher risk of prostate cancer [51]. The rs2294008 variant is a variant that not only has no potential role in prostate cancer (prostate carcinogenesis) has no association with PSA, GS, Gleason score, age, stage and metastasis in prostate cancer [53].

### PSCA in breast cancer

Breast cancer is one of the most common cancers and the second leading cause of death in women in the world. Histological types of this cancer include invasive lobular carcinoma (ILD), invasive ductal carcinoma of nonspecial type (IDC-NST), invasive micropapillary carcinoma (IMPC) and 23 other types [67, 68]. Conventional diagnostic methods for this cancer, which include mammography and ultrasound imaging, are not accurate and sensitive enough to diagnose this cancer, and this can be backup by the reports that the age distribution of this cancer is relatively in the young people who have dense breasts and the lesion will be difficult to be diagnosed [68, 69].

One of these new biomarkers, which was identified using the microarray gene expression profiling analysis method and has a distinct expression in breast cancer (IMPC-IDC-NST), is PSCA [70]. IHC confirmed that PSCA is not expressed in normal breast tissue or stroma cells, but in breast cancer tissue, its distribution in the membrane and sometimes in the cytoplasm is evident. In breast cancer, the lowest expression is seen in a few disseminated tumor cells and the highest expression is seen in invasive tumor cells [54].

The expression of PSCA in breast cancer seems to be related to clinicopathological features so that the expression of this protein is associated with undesirable histopathological grade and increased proliferation activity and has no association with age, histological subtype, tumor stage, nodal status, lymphatic-invasion or angio-invasion and survival of patients [54]. Also, there is no correlation between PSCA protein expression and estrogen and progesterone receptor status, but there is a strong correlation between this protein expression and Her2/neu receptor status, explaining that breast cancer patients with high PSCA protein expression are more likely to be associated with overexpression of Her2/neu [54]. The expression of PSCA is different in histological types of breast cancer, so the highest expression is in IMPC and NST-IDC, and the lowest expression is in ILC [70]

In IMPC, PSCA is involved in cluster cell metastasis and high PSCA expression is associated with the lowest disease-free survival (DFC) compared to IMPC patients in whom PSCA expression is negative. Finally, the function of PSCA in breast cancer is not fully understood, however, findings suggest that PSCA is more likely to act as an oncogene than as a tumor suppressor. PSCA undergoes gene amplification in breast cancer and may be associated with protein overexpression [70].

### PSCA SNPs in breast cancer

Numerous studies have shown that the susceptibility to cancer in some organs is associated with the 8q24 locus, such as prostate, gastric, bladder, etc. The PSCA gene is located downstream of the 8q24 locus and away from breast cancer. Studies have suggested that PSCA SNPs are associated with breast cancer risk. Among the many PSCA SNPs, rs2294008C > T, rs2978974G > A and rs2976392G > seem to have the highest number of breast cancer studies in the Chinese population. The findings of Wang et al. [71] show that both the homozygous model and the recessive model of rs2294008 are associated with breast cancer, but the other two SNPs were not significantly associated with breast cancer, and there was also no association between age and this SNPs in this cancer. In terms of menopausal status, the minor allele rs2294008 is an important risk factor in both premenopausal and postmenopausal women, and the heterozygous rs2978974 model is an important risk factor in postmenopausal women, while the rs2976392 allele has nothing to do with the menopausal status in Chinese women. Numerous studies have been performed on the association of these 3 SNPs with clinicopathological characteristics. The results show that rs 2294008 T is associated with progesterone receptor status and rs2976392 minor allele is associated with lymph node metastasis and rs2978974 allele has no association with clinicopathological characteristics. Finally, analysis of PSCA haplotype showed that TAG haplotype is associated with a significant increase in breast cancer risk [71]. In addition to the above SNPs, rs2976395 is found as a risk factor for estrogen receptor tumors [72].

### PSCA in brain cancer

Reports from the World Health Organization indicate that the death rate from brain cancer in Asia is very high. Brain cancer occurs in the brain or nerve cord and is divided into different types depending on the nature, origin, growth rate, and progression and can be malignant or benign.

Numerous studies have shown that PSCA protein is expressed in the brains of mammals and birds, but its expression in normal brain tissue is not reported except in Glioma, which could be used as a target therapy [73]. Other research showed that PSCA expression was present in normal neural and plexus cells of the human brain, as confirmed by the RT-qPCR method in which a low expression level of PSCA occurs. The presence of PSCA was also confirmed by IHC, using mouse anti-PSCA antibody to evaluate its expression in normal brain cells [74].

In brain tumors, PSCA is upregulated in meduloblastoma, glioma, papilloma and papillary carcinoma of choroidplexus, ependymoma and meningioma. The function of PSCA in the brain is unclear, but its function in the neuronal cells of chick peripheral ganglia acts as an antagonist against inducing cell death and thus rescuing neuronal cells. As the ganglion expands, the expression of PSCA expands accordingly [75]. The results of the study of PSCA expression in the brain suggest that PSCA has a tumor-promoting role in brain tumors and can be a therapeutic target for it, but may have adverse effects on the central nervous system [74].

PSCA also appears to play a role in the development of Alzheimer's. Alzheimer's is a neurodegenerative disorder caused by the degradation of cholinergic neurotransmission and dysregulation of nicotinic acetylcholine receptors (nAChRs) and changes in a4b2 nAChR levels [76, 77] mediated by the superfamily members of the Ly-6/neurotoxins or (Lynx) proteins. PSCA is one of the Lynx proteins, which suppresses nicotine-induced calcium infiltration by activating 7α nACHRs when expressed retrovirally in ciliary ganglion neurons [75]. Also, a study with recombinant PSCA demonstrated that PSCA and a4 nAChR subunit form a stable complex in the human cerebral cortex and prevent nAChR signaling and cause its dysregulation in the frontal cortex of AD patients [77]. In patients with Alzheimer's disease, the level of PSCA protein in the frontal cortex increases, this in turn decreases the function of nAChRs in these patients, so PSCA-nAChR interactions may be effective in cognitive function, especially in AD pathology [78].

### PSCA in gastric cancer and gastric ulcer

Gastric cancer is one of the fifth most common cancers worldwide and the third leading cause of cancer death [79]. Factors involved in the etiology of gastric cancer include *Helicobacter pylori (H.pylori)* infection, environmental factors, genetic susceptibility, diet, smoking, pernicious anemia and Epstein-Barr virus and E-cadherin gene [80].

GC is divided into two types based on localization: Cardia and Noncardia, and histologically includes Intestinal and Diffuse types. Studies by the Genome-Wide Association Study (GWAS) have shown that 5p13 and 8q24 risk loci are involved in the etiology and progression of gastric cancer in the European population [72, 81, 82]. In vitro studies have shown that PSCA mRNA is expressed in normal gastric mucosa tissue and mainly in the isthmus of gastric glands, while is absent in gastric tumor tissue. Also, PSCA protein is expressed in gastric neuroendocrine cells [13, 46, 82]. PSCA is one of the down-regulated genes in gastric cancer, and has a low expression level in GC, Thus PSCA is a tumor suppressor in gastric cancer and prevents cell proliferation [55], but its mechanism of action is unknown.

PSCA polymorphisms are also involved in the development of gastric cancer. GWAS has identified the role of rs2294008 and rs2976392 SNPs in GC in Chinese, Japanese, Korean, and Caucasian populations, and these SNPs appear to alter the physiological properties of PSCA and affect its expression level. Based on a study, rs2294008CT and rs2294008CT + TT can increase the risk of gastric cancer in the Chinese population (in young people, non-smokers, non-alcoholics, men and non-cardia patients). The possible mechanism of action for this polymorphism is that this variant reduces the transcriptional activity of the PSCA upstream fragment [56, 83]. It was found that the rs2294008 T allele resulted in a significant reduction in transcriptional activity of the PSCA promoter in gastric cell lines [32]. Another mechanism of action for this variant may be its effect on proinflammatory and anti-inflammatory signals in gastric mucus [82].

Another function of allele T rs2294008 is related to the activation of YY1, so that the activation of YY1 in prep pit cells in individuals carrying the T allele leads to the extinction of PSCA silencing, and the T allele recruits YY to its promoter which suppresses PSCA copying activity and GC occurrence [84].

The rs2976392G > A variant is also associated with an increased risk of gastric cancer, but its mechanism of action is unclear. This variant has a strong linkage disequilibrium with rs2294008, which could justify its function [83]. rs2294008 variant is more associated with diffuse-type gastric cancer than intestinal type in Korean and Japanese populations. Rs2294008T variant is more associated with diffuse type in Asian population than Caucasian population due to the high T allele in this population. Also, the homozygous type of these two variants, rs2294008 and rs2976392, have a higher percentage of susceptibility to gastric cancer than the heterozygous type [32, 85]. On the other hand, CA haplotype between rs2294008C/T and rs2976392A/G is associated with a significant reduction in gastric cancer in Tebyan population [86]. The role of rs2294008 and rs2976392 in the incidence of cancer in Uzbekistan has also been investigated and the results indicate that the rs2294008 T allele is associated with an increased risk of GC and rs2976392 located in intron 2 has a significant association with GC in women under 40 years of age [87].

It has previously been suggested that one of the regulatory mechanisms of PSCA is estrogen receptors, because the ER-binding site is located in the promoter region of the PSCA gene and regulates its expression [88], and young women, for they have more estrogen, are more likely to be affected by rs2976392 variant and thus GC risk87. Another study evaluated the association between rs2294008 and *H.pylori* and concluded that *H. pylori* infection has a profound effect on T allele carriers and suppresses PSCA expression, which is abundant in normal mucosal tissue [89]. Also, examining the association between PSCA polymorphism and the prevalence of intestinal metaplasia (IM) in *H. pylori*-infected Bhutanese reported that patients carrying the TT genotype were three times more likely to develop IM than those carrying C [90].

PSCA antigen is also associated with gastric ulcer, so people who carry the C allele variant rs2294008 are more susceptible to gastric ulcer. The mechanism of action can be explained by the fact that the T allele of variant rs2294008 produces a protein that has an extra 9 amino acid fragment in the N-terminal region of the PSCA protein [31] while the C allele produces a shorter protein. Studies have shown that the shorter protein remains in the cytoplasm, lacks the C-terminal region and the glycosylation site, and therefore is more rapidly affected by proteosomal degradation. This short protein also activates CD4 or CD8-positive T cells and thus epithelial mucosal injury increases, but full-length protein on the surface of the cytoplasm improves the repair of epithelial mucus by enhancing cell proliferation [91, 92].

### PSCA in colorectal cancer (CRC)

Colorectal cancer is a cancer that develops in the colon or rectum. This cancer typically affects the elderly, but it can occur at any age [93]. CRC usually first appears in the colon as small masses of noncancerous (benign) cells called polyps. Over time some of these polyps may develop into colon cancer. Colorectal cancer is one of the most common cancers. Genomic and epigenetic alteration, environmental and genetic factors are effective factors in the development of CRC [93–95].

IHC analysis showed that the color intensity was higher for PSCA protein expression in neuroendocrin cells, but colonocytes were weak or negative for PSCA protein expression. There were no changes in topographic distribution between normal mucosa and adenomatous mucosa with low or high dysplasia and the incidence of carcinoma invasion [63].

Rs2294008 SNP, which is a polymorphism of PSCA, may also play a role in the development of CRC, although there are conflicting results. As TT allele is much higher than that of CC in CRC patients, and CC carriers are associated with an increased susceptibility to CRC compared to T carriers. Stratification analysis of parameters such as age, gender and smoking have shown the increased susceptibility to CRC by TC/TT or CC allele. In addition, TT and CT genotypes showed a poor prognosis compared to CC genotype, and this allele was associated with poor survival of CRC patients compared to CC [96]. In both Chinese and European populations, PSCA polymorphism is associated with an increased susceptibility to CRC and a poor prognosis [97]. Other research found no relationship between PSCA and its polymorphism, rs2294008, with the incidence, onset and progression of CRC [63].

### PSCA in duodenal ulcer (DU)

Duodenal ulcer is one of the most common gastrointestinal disorders and the spread of ulcer, occurs during a multi-stage process. One of the factors involved in DU can be infection with *H. pylori*, resulting in damage and inflammation of the duodenal mucosa.

GWAS studies on the Japanese population have identified the role of two gene loci in the development of DU: PSCA on chromosome 8q24 and ABO on chromosome 9q34. To understand the role of these two loci, the expression of PSCA and ABO in the duodenal epithelial cell was examined. ABO genes were found to be expressed in the gastrointestinal tract, including the duodenal epithelium, but the PSCA gene is not expressed in the normal duodenum and may be expressed in the duodenum of gastric-type mucous secreting cells in the duodenal ulcer [31].

The two alleles of the PSCA rs2294008 genetic variant have different effects on the incidence of DU and GC, so the T allele reduces the susceptibility to DU and increases the risk of GC. The C allele is the opposite and increases the susceptibility to DU and the results indicate that the rs2294008 genetic variants affect the duodenum with growth-promoting effect by T allele and effect on T-cell activation by C allele [31].

The hypothesis for the function of rs2294008 explains that when the duodenal mucosal cells are damaged, the tissue repair system responds by platelet aggregation, releasing growth factors. Subsequent proliferation and migration of epithelial cells occur and eventually lead to tissue repair, however, people with homozygous C allele and a cytoplasmic form of PSCA, lack functional PSCA, and thus epithelial proliferation is not performed enough in these patients and duodenal tissue damage occurs slowly. Also, individuals with short PSCA protein, are more susceptible to degradation by the proteasomal degradation system, supplied by HLA molecules, and activation of CD4 + /CD8 + T cells, which inhibit tumor growth but cause peptide ulcers [31].

The relationship between the index and various parameters, such as the relationship of rs2294008PSCA with age, sex, smoking, drinking and others is also examined. Results indicated that smoking, drinking and infection with *H.pylori* and even coffee consumption do not show a special relationship between rs2294008 and DU, yet rs2294008 SNP can still increase the susceptibility to DU, and logistic regression analysis showed that age, gender, and smoking are independent risk factors for DU [30, 31].

### PSCA in esophageal squamous cell carcinoma (ESCC)

Esophageal cancer is one of the deadliest diseases due to its aggressive nature and also the very low survival rate, ranking the eighth most common cancer among all cancers. Risk factors include smoking and hookah [98], alcohol and tobacco consumption [99] drinking hot tea, high consumption of red meat, low absorption of fresh fruits and vegetables, obesity and other factors [100].

Esophageal cancer consists of two subtypes: Squamous cell carcinoma and Adenocarcinoma [101]. Northern blot analysis has shown that PSCA is expressed in esophageal epithelial cells, but in undifferentiated esophageal tumors the PSCA mRNA and its protein are reduced to such an extent that even sometimes it is unrecognizable [102].

**Fig. 2:**
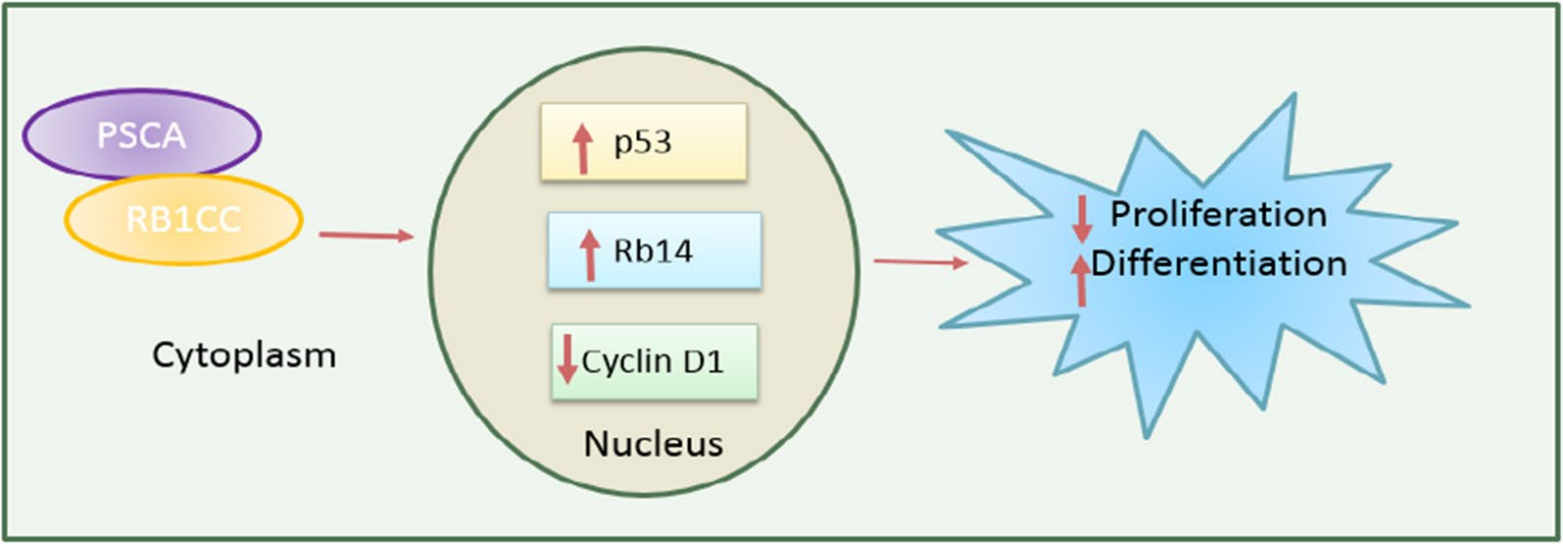
PSCA controls cell cycle and differentiation regulators through modulating RB1CC1 protein stability and nuclear translocation. The binding of PSCA to RB1CC1 in cytoplasm results in stabilization and translocation of RB1CC1, which in turn increases levels of p53, Rb14 and decreases cyclin D1. As a result, proliferation decreases while differentiation increases

The results of studies on the relationship between PSCA expression and clinicopathological characteristics show that PSCA down-regulation is associated with poor differentiation, lymph node metastasis and advanced stage. Also, patients with ESCC with PSCA down-regulation, have shorter disease-specific survival compared to patients with normal PSCA expression. Numerous reasons for PSCA down-regulation were examined in the ESCC, such as deletion or hyper-methylation of the promoter. The results showed that the site where the PSCA is located, in the 8q24 chromosome, is amplified in more than 75% of the ESCC and CpG was not observed in the promoter of this gene in ESCC. The effect of T allele rs2294008 polymorphism on PSCA expression was investigated which showed no association with PSCA expression. Finally, the effect of TF SOX5 on the down-regulation of PSCA was investigated, which indicated SOX5 binding site 2.5 kb upstream of the PSCA gene transcription start site directly suppresses PSCA expression. PSCA controls the cell cycle and differentiation regulators, by modulating the cytoplasmic stability of retinoblastoma 1-inducible coiled-coil 1 (RB1CC1) and facilitating its nuclear translocation **(**Fig. 2**) **[62].

The rs2294008 polymorphism also plays a role in the development of ESCC, as it reduces the risk of esophageal cancer in people carrying T, CT and TT alleles compared to those carrying CC alleles. The relationship between rs2294008 and parameters such as age, sex, smoking and alcohol consumption is also investigated, which showed a strong association between all gene types and ESCC risk in alcohol drinkers [103].

### PSCA in pancreas cancer (Pac)

Pancreatic cancer is the fourth leading cause of death in developed countries. Risk factors for this cancer include smoking, type 2 diabetes mellitus, chronic pancreatitis, and genetic factors. This type of cancer has a poor prognosis and is diagnosed when the cancer is very advanced. Due to the lack of specific and sensitive markers to identify cancer, it is necessary to look for target candidates for diagnosis and treatment.

PSCA is not expressed in the normal pancreas, which was confirmed by various tests such as Northern blot and IHC, however, research using IHC and double-staining methods showed that PSCA expression is seen in all 4 types of normal human pancreatic islets (α, β, δ and PP cells). Also, by studying the start position of PSCA transcription, two separate variants of PSCA in the pancreas are identified [15, 104]. One is variant I, which is identified in other tissues, and the second type is non-coding type or variant II, which is located at the start site of transcription and 1 Kb upstream of variant I, and states that the regulatory position of variant II is different from type I and its size is small and does not encode any protein, however, variant I is predominant in both normal and tumor tissue [15].

The expression pattern of CD44 and PSCA in pancreatic cancer showed that positive expression of PSCA was associated with tumor size and nodal metastasis, but its expression had no association with tumor differentiation [57]. PSCA is up-regulated in pancreatic cancer, and its IgG antibodies are present in the plasma of patients with pancreatic cancer. About 57 types of peptides are encoded by the PSCA, of which antibodies to these peptides are significantly higher in the blood of people with pancreatic cancer (10 out of 57), and the measurement of these antibodies can provide new ways to diagnose and treat this type of cancer [58].

### PSCA in gallbladder cancer (GBC)

Gallbladder cancer is a relatively rare cancer but has a high mortality rate. There are several risk factors such as genetic changes involved in the development of this cancer. PSCA seems to play a role as a new marker in the development of this cancer. Northern blot method revealed that PSCA is expressed in the normal gallbladder [102]. Also, IHC analysis showed its localization in the epithelial cells, mainly in the cytoplasm and homogeneously throughout the epithelium, in addition to epithelial cells, it is also expressed in gallbladder smooth muscle cells [105]. PSCA is also expressed in gallbladder cancer, so its expression in gallbladder cancer with adenocarcinoma is much higher than pericancerous tissues, polyp, chronic cholecystitis of the gallbladder epithelium and its expression in gallbladder epithelium with mild hyperplasia or in normal tissue is very low [106]. PSCA acts as down-regulation in this cancer, as in cholecystitis, mucinous adenocarcinoma, adenosquamous cell carcinoma, undifferentiated carcinoma, adenomyomatosis, and metaplasia its expression is down-regulated, but it is not clear for cholesterolosis or in polyp [105].

The association analysis between PSCA and the clinicopathological features of gallbladder cancer showed that PSCA overexpression is related to tumor differentiation, lymph node metastasis, T-stage, and OS implying that PSCA could play a vital role in carcinogenesis and tumor progression. Meanwhile, its expression has no association with parameters such as age, sex and history of gallstones [106].

The possible role of DNA methylation on PSCA gene and androgen receptor (AR) regions in the mechanism of action of PSCA down-regulation in GBC was investigated by bisulfite-pyrosequencing method. The results showed that the rate of DNA methylation is inversely proportional to PSCA expression. And AR that is located in the enhancer region of PSCA has no role in the regulation of PSCA expression in the GBC. PSCA in GBC cell line prevents cell proliferation and reduces tumor invasion, thus it elicits a tumor-suppressor action [105].

GWAS studies have identified PSCA polymorphisms, rs2294008 and rs2978974, as genetic risk factors for GBC cancer. The rs2294008T allele, located in exon 1, affects PSCA transcriptional activity. Since PSCA acts as a tumor suppressor in the GBC, therefore, decreased PSCA anti-tumor activity, increases the risk of developing this cancer [84, 107, 108]. On the other hand, various studies have shown that there is no significant association between PSCA polymorphisms and the incidence of GBC cancer. Also, statistical analysis showed the association of Trs2294008-Grs2978974 haplotype in increasing the incidence of gallbladder cancer in women, while the haplotype (Trs2294008 -Ars2978974) has the lowest risk of GBC in men [109]. As the level of PSCA expression in women is lower than in men, thus reducing the level of PSCA protein and consequently reduced antitumor activity will have a greater effect in women [110]. Another reason to justify the different performance of rs2294008 and rs2978974 variants among men and women can be explained by the androgen receptor binding site and (ARE) androgen-responsive element [11]. Such that, the loss of these regions due to changes caused by PSCA polymorphisms and decreased androgen levels, can change the mechanisms of action of PSCA in men and women [109].

The mechanisms by which the T allele of variant rs2294008 suppresses PSCA transcriptional activity in GBC cancer is explained by the notion that the T allele attracts the Ying Yang1 (YY1) transcription factor to the PSCA promoter by creating a consensus binding site and suppresses the transcription of the PSCA gene, thus reducing the number of PSCA copies and subsequently causing GBC [84].

### PSCA in nasopharyngeal carcinoma (NPC)

Nasopharyngeal cancer is one of the cancers of the head and neck squamous cell carcinoma (HNSCC) and the two main characteristics, distant metastasis and local recurrence, are the causes of failure in the treatment of this cancer. Hopefully, PSCA antigen is one of the markers involved in this cancer and can serve as a new therapeutic marker.

IHC staining has shown that PSCA is present in the cytoplasm and membrane of nasopharyngeal carcinoma cells (NPCs), and acts as down-regulation. Low PSCA expression is seen at both protein and mRNA levels in NPCs with the most metastasis compared to NPCs with the least metastasis [60]. The relationship between PSCA protein expression level and physiological characteristics demonstrated that low PSCA expression is associated with distant metastases with no association with parameters such as age, sex, T stage or N stage and a high expression of PSCA associates with longer overall survival (OS) and distant metastasis-free survival (DMFS) in patients with nasopharyngeal cancer [60].

**Fig. 3:**
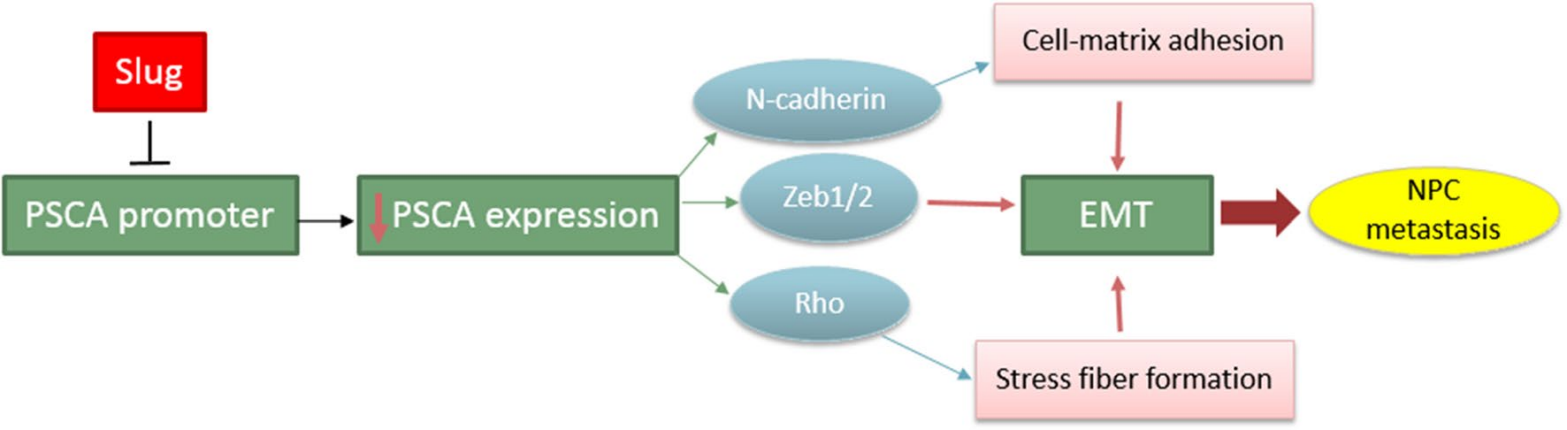
Proposed mechanism for regulation and function of PSCA by slug in NPC incidence. The binding of the slug to PSCA promoter causes PSCA downregulation and consequently N-cadherin and ZEB1/2 increase. These changes activate Roh A, which initiates the EMT induction process and causes nasopharyngeal cancer

PSCA acts as a negative regulator for migration and invasion of NPC cells, as PSCA knockdown increases these two parameters, while PSCA overexpression abrogates these effects in vivo. The mechanism of action is that the PSCA may negatively modulate the metastatic process in NPC cells by programming epithelial-mesenchymal transition (EMT) process [60]. PSCA expression is suppressed by Slug transcription factor, which is involved in TGF-β-dependent EMT and NPC metastasis [111, 112]. Two E-box motifs in the PSCA promoter are the junction of Slug and Snail. Slug junction to this motif causes down-regulation of PSCA and subsequently increases N-cadherin and ZEB1/2, activating RohA, which initiates the EMT induction process and causes nasopharyngeal cancer. Therefore, PSCA can be considered as an independent prognostic factor for patients with nasopharyngeal cancer **(**Fig. 3**) **[88].

### PSCA in oral squamous cell carcinoma (OSCC)

OSCC is one of the most common oral cancers caused by environmental and genetic factors [113, 114]. PSCA is transcribed differentially in normal and cancerous head and neck squamous tissue, and that expression of PSCA mRNA and its protein in OSCC is much higher than that of its normal counterpart. Also, IHC staining confirms the presence of its protein and mRNA in the cytoplasm of OSCC cancer cells but its expression in normal tissue is low and expressed in keratin epithelial cells, however, different genotypes are involved in the localization of PSCA protein [115, 116].

The mechanism of regulation of PSCA expression in this cancer is not known, but considering H314 and H413 cells, which are both oral cancer cells and are p53 mutants in different positions, this can affect PSCA expression through complex signaling pathways [116].

In addition to PSCA dysregulation, its very common polymorphism rs2294008 is also involved in the development of OSCC cancer. The frequency of rs2294008CC genotype is higher in patients with OSCC and indicates a predisposing factor for this cancer. As mentioned earlier, different genotypes are involved in the localization of PSCA protein. That, T allele of variant rs2294008 changes the position of the protein from the cytoplasm to the cell surface, and in patients with CC genotype, the cytoplasm of epithelial cells and nests are PSCA-positive, while in patients with CT/TT allele, epithelial cornification membrane and the cytoplasm of basal epithelial cells and nests are PSCA-positive [31, 116]. Moreover, a study on the relationship between rs2294008 variant alleles and clinicopathological characteristics showed that patients carrying CT/TT allele are associated with tumor size, stage, and lymph node metastasis but are not significantly associated with cancer recurrence and may have the opposite effect based on the survival curve [116]. Finally, PSCA and its rs2294008 polymorphism may be involved in the development, progression and prognosis of OSCC, but the mechanism is not yet clear.

### PSCA in non-small cell lung cancer (NSCLC)

Lung cancer is a type of cancer that causes death and the most common type is NSCLC. PSCA has provided new hope in the diagnosis and treatment of this type of cancer [117, 118].

Data from IHC analysis indicated that the PSCA protein is not present in the epithelium of normal lung tissue but is found in its cancer counterpart and is localized in the cytoplasm of lung tumor cells. PSCA is also associated with pathological T factor and tumor stage so in metastatic NSCLC, PSCA expression is very high [59].

A possible hypothesis about the mechanism of action of PSCA in lung cancer is that C-myc, which is one of the proteins involved in lung cancer, is one of the factors influencing PSCA expression [59]. PSCA in NSCLC is more likely to develop overexpression [59]which can be due to co-amplification with C-myc oncogene[46]. When PSCA is knocked down, the growth of human lung tumor cells is inhibited, so it can be concluded that PSCA is one of the proteins involved in the development and metastasis of lung cancer[59].

### PSCA in endometrial adenocarcinoma (EAC)

Endometrial cancer is one of the most common cancers in obstetrics and gynecology and its main causes are obesity, high blood pressure, hormonal changes, and increased estrogen levels.

The PSCA marker is not present in normal endometrial tissue, but IHC analysis confirmed the localization of PSCA protein in the cytoplasm of endometrial tumor cells. In endometrial cancer, the mechanism by which PSCA expression occurs is not known, but it is suggested that T-Box Transcription Factor 2 (TBX2), one of the markers involved in endometrial cancer, may play a role in up-regulating PSCA expression [119].

In the EAC, PSCA expression is found to be associated with age, tumor grade, and lymph node metastasis but has no association with the pathological stage. As PSCA expression is much higher in people over 54 years of age than in people under 54 years of age, its expression also increases with increasing pathological grade and lymph node metastasis. However, since PSCA has no association with tumor stage, it may have a greater role in tumor initiation than in invasion [119]. Thus, PSCA can be a biomarker and therapeutic target for screening patients over 54 years of age in the EAC due to its association with age, tumor grade and lymph node metastasis.

### PSCA in cervical adenocarcinoma

Cervical cancer is a type of cancer that leads to death and the main cause is persistent human papillomavirus (HPV) infection. PSCA is one of the biomarkers that may be involved in cervical cancer. IHC confirmed the presence of PSCA in the cytoplasm of cervical tumor cells, although different genotypes can affect the localization of PSCA in cervical tumor cells [120]. PSCA acts as an up-regulation in cervical cancer and its expression is related to the rate of cancer invasion, thus PSCA plays an oncogenic role in cervical cancer, but the mechanism of action is not clear [61].

PSCA polymorphisms are also involved in the development of this cancer. The TT allele of variant rs2294008 is associated with a reduced susceptibility to cervical cancer. The association of the TT allele with parameters such as pre-menopausal and post-menopausal has also been investigated and the results indicate the predominance of these alleles, and PSCA is a valuable biomarker, especially for early-stage cervical cancer. Mechanistically, the T allele reduces the transcriptional activity of the upstream fragment of the PSCA gene, resulting in reduced PSCA transcripts and subsequently reduced PSCA protein and ultimately reduced PSCA oncogenicity in cervical cancer. In addition to rs2294008 variant, two other PSCA variants (rs2976392, rs2978974) were evaluated in the incidence of cervical cancer, but whether these two variants are involved in cervical cancer needs further investigation [120].

### PSCA in gestational trophoblastic disease (GTD)

Gestational trophoblastic disease is a term for a group of tumor-causing diseases that originate in the placenta. These tumors are very rare and occur when placental cells grow out of control. There are different types of this disease, of which hydatiform mol (HM) and choriocarcinoma (CCA) are the main forms of this disease [121].

IHC analysis has shown severe irritability of the cytoplasm to PSCA in all types of trophoblasts, as seen in extravillous trophoblasts and HM, but at various expression levels. In that, its expression in the original natural placenta is much less than HM, mild to moderate expression is seen in the spontaneously degraded HM type, and the highest expression of PSCA mRNA is seen in HM type, which leads to the development of gestational trophoblastic neoplasia. The most severe mRNA PSCA expression is choriocarcinoma and the placental site of trophoblastic tumor. PSCA is overexpressed in the persistent hydatiform mole and may be one of the factors that enhances the conversion and development of HM to the GTN type, but how PSCA works in HM needs further study [66]. PSCA is shown to elicit a direct role in cell death [122, 123] and HM with high apoptotic indexes tend to decay, and apoptotic activity is effective in the progression of HM to GTN [124, 125].

PSCA is associated with proliferation indices such as Ki67, MCM7, p53, P21, mdm2 and M30, supporting the theory that PSCA is involved in regulating cell cycle progression in trophoblast tissue. The positive correlation between PSCA and mdm2, p21^WAF1/CIP1^ shows that wherever PSCA expression increased, the expression of p21^WAF1/CIP1^ and mdm2, and trophoblastic growth is increased [66].

### PSCA in clear cell renal cell carcinoma (CC-RCC)

RCC is a heterogeneous cancer and CC-RCC is a very common type, divided into various types based on physiological properties [126]. Several biomarkers are involved in the development of this cancer, and PSCA is one of them. PSCA is expressed in both normal kidney tissue and tumor tissue, but the level of expression varies. IHC analysis shows that PSCA is visible both on the cell surface and cytoplasm of the normal kidney [64]. Staining using anti-PSCA has shown that PSCA expression in normal tissue is very low due to its poor staining in collecting ducts and some tubules and is not expressed in normal glomeruli cells [46]. Analysis of RT-PCR data showed that the level of PSCA mRNA expression in renal tumor tissue was much higher than normal tissue. Also, the relationship between PSCA and clinicopathological features showed that PSCA mRNA expression level in G2-G3 tumors was much higher than G1, and in advanced tumors, that is, in T3-T4 it is much higher than T1-T2 and in M1 it is more than M0. Findings indicated that PSCA in kidney cancer is associated with tumor grade, tumor stage and tumor progression but not specifically with parameters such as age and sex. PSCA in CC-RCC has an up-regulation function and because it is associated with the degree of tumor differentiation, it plays a key role in the progression of this cancer. Its high expression in the advanced type shows its role in CC-RCC tumor invasion. In addition, patients with CC-RCC cancer who have high PSCA expression have worse cause-specific survival than patients with low PSCA expression [64]. Thus, PSCA can serve as a new biomarker in the diagnosis, treatment and prognosis of advanced-stage CC-RCC.

### PSCA in bladder cancer

The bladder is a hollow organ with flexible muscle walls, and bladder cancer is the fifth most common cancer in the United States, and the fourth most common type of cancer in men, and women, accounting for one-third of men [127]. Bladder cancer can be divided into invasive and non-invasive in terms of its spread in the wall and papillary or flat in terms of growth. There are several types of bladder cancer, the most common of which is transitional cell carcinoma (TCC).

Numerous studies have identified the role of PSCA as a new marker in the development of bladder cancer. The PSCA expression pattern suggests that PSCA protein is expressed in both normal tissue and tumor type. Northern blot analysis revealed low PSCA mRNA expression in normal bladder tissue, and higher in the normal bladder urothelium. IHC staining also showed its localization in normal tissue is limited to the superficial umbrella cell layer. Also, confocal microscopy showed PSCA protein expression at the cell surface using 1G8 mAb [65, 102].

PSCA mRNA is expressed in bladder tumors, including superficial transitional cell tumors (STCC), muscle-invasive TCCs (ITCCs), invasive transitional cell, carcinoma in situ (CIS), and even the dysplastic type, and its expression is much higher than normal tissue. In terms of expression level, it has the highest expression in CIS and superficial tumors, but in more than 30% of invasive and metastatic muscles, the expression level of PSCA also increases significantly. The subtle expression is seen in the transitional epithelium of normal bladder tissue. It is important to note that in normal and dysplastic tissue, PSCA is expressed in the superficial layers of the urothelium but in the CIS it can be detected in all neoplastic urothelium layers. In both STCC and ITCC, higher PSCA expression level correlates with higher tumor grade [65].

The relationship between PSCA expression in bladder cancer and parameters such as tumor grade and tumor differentiation indicates that poorly differentiated non-muscle invasive tumors have a higher expression level than differentiated superficial tumors [65]. Conversely, other studies stated that PSCA mRNA is dramatically reduced or even unrecognizable in undifferentiated bladder tumors [102]. And that PSCA expression is inversely related to tumor stage [65, 128].

Together, the function of PSCA in CIS and non-muscle invasive tumors and muscle-invasive tumors is mostly overexpression possibly due to PSCA gene amplification confirmed by fluorescent in situ studies [65] based on the cell-cell relationship and surface adhesion [102].

A multivariate analysis examined PSCA mRNA expression levels and their association with age, sex, disease recurrence, stage and grade of the tumor, and reported that PSCA expression was an independent prognostic factor for assessing tumor recurrence. In superficial TaT1 tumors, the level of PSCA expression in recurrent superficial TCC is much lower than in patients without recurrent TCC, and the recurrence of the disease has no special relationship with any of the aforementioned parameters [128].

Additionally, GWAS studies have identified the role of rs2294008 T allele in increasing PSCA mRNA expression in normal bladder and tumor tissue. It is stated that rs2294008 alters the length of the PSCA N terminal signal peptide, thereby altering the behavior and localization of the PSCA. rs2294008 is also reported to be an independent locus in bladder cancer [129]. Another study indicated that the level of PSCA mRNA expression is higher in patients with bladder cancer who carry the T allele compared to patients with the homozygous CC allele [130]. Moreover, ethnicity studies, have shown that rs2294008 has an increasingly effective role in the development of bladder cancer among Asian and Caucasian populations [131] with rs2294008 (C > T) polymorphism in US and European populations [129].

A meta-analysis confirmed the role of rs2294008 polymorphism in bladder cancer in European, North American and Asian populations. That rs2294008 has allele-specific effects on (protein and mRNA) PSCA expression. And, due to allelic imbalance, functional changes of protein and PSCA mRNA occur as a result of the acquisition of rs2294008T allele and loss of rs2294008 C allele. In bladder cancer, with increasing tumor stage, the level of PSCA expression decreases due to a decrease in the expression of the non-risk C allele, while the T allele of PSCA expression is not associated with tumor stage. Finally, at least 75% of European patients with bladder cancer carry CT, TT alleles of rs2294008 genotype [132].

Another PSCA polymorphism, rs2736098, is likely to be associated with an increased risk of bladder cancer, as the T allele of this variant is associated with an increased risk of bladder cancer compared to patients carrying homozygous C allele [131].

## PSCA-based cancer theranostics

PSCA-based theranostics platforms can be used as a new biomarker as well as a therapeutic agent for different types of tumors. Compared to routine methods such as IHC, PSCA-conjugated nanoparticles, provide faster and sensitive prognostic and diagnostic tools.

In our previous study, PSCA antigen was conjugated with superparamagnetic iron oxide SPIONs. Acknowledging that PSCA antigen has a high expression in high grades of prostate cancer and thus the intensity of the color produced in the IHC test is also higher. Our results showed the high color intensity of iron staining in high grades of cancer. PSCA-conjugated SPIONS is a new method to detect prostate cancer, which is faster, safer and cheaper than the conventional IHC method [133]. In another study, multiwalled nanotubes (MWCNTS) were conjugated with PSCA monoclonal antigen and used for prostate imaging. The results showed that CNT-PE(FITC) mAb can be used for simultaneous imaging and drug delivery at the same time [134].

Dual-function nanoparticle polymer composed of scab-PLGA_SPIONs/docetaxel specifically binds to the PSCA antigen in prostate cancer cells, and promotes accumulation, uptake, cellular absorption and thus the intracellular delivery of the docetaxel drug. This polymer-nanoparticle simultaneously increased drug delivery performance and enhance the resolution of MRI in prostate cancer [135].

A multimodal immunotheranostic involving IgG4-based anti-PSCA antibody (anti-PSCA IgG4-TM) conjugated with the DOTAGA chelator is developed which is capable of positron emission tomography (PET) diagnostic imaging, targeted alpha endoradiotherapy, and chimeric antigen receptor (CAR) T cell immunotherapy. A single injection of the ^225^Ac-labeled anti-PSCA IgG4-TM effectively localized antibody to target cancer cells expressing PSCA, promote tumor lysis and significantly control tumor growth in mice [136].

It is shown that A11 minibody strongly binds to PSCA antigen. Therefore, it can be used to diagnose and treat prostate cancer at once. For this purpose, A11 minibody conjugated gold nanoshells (A11-conjugated gold nanoshells) were used for photodermal therapy of prostate cancer. This formulation benefited the gold ability of laser-induced heat generation and localized killing of prostate cancer cells [137].

Finally, the polyplex system, which is designed based on the combination of siRNA with siRNA-mediated survivin knockdown and scFv-madiated PSCA inhibition, is used for targeted delivery. In this study, BIRC5/Survivin RNAi using propylene imine conjugated antibody prevented the growth of tumor cells that express PSCA antigen on their surface [137].

## Discussion

PSCA is an antigen that is expressed in more than 80% of prostate cancers. In addition to PSCA, the prostate has another antigen called PSA, which, unlike PSCA, is mainly expressed in normal prostate tissue, while the expression of PSCA in normal tissue is very low compared to prostate cancer tissue, therefore, thus this antigen can be used as a new marker in differentiating tumor tissue from normal glands during prostatectomy [1]. On the other hand, this antigen is a protein that binds to the cell surface through the GPI interface, so its cell surface location can serve as a target for the development of cancer diagnosis and treatment platforms (theranostics). That is, anti-PSCA antibodies conjugated to the NPs, can carry fluorescent probes, radioisotopes, toxins, and chemotherapy drugs, to realize localized cancer cell targeting, imaging and therapy [138]. PSCA-based antibodies especially those based on CAR T cell immunotherapies are more popular and several are undergoing clinical trials (Table 6).

**Table 6:** PSCA-based immunotherapy in preclinical and clinical settings

Method	Major findings	Reference/NCT
DOTAGA-conjugated anti-PSCA IgG4-TM	Multimodal theranostic platform uses uniCAR T cell for *in vivo* imaging and immunoradiotherapy of prostate cancer.	[136]
I^131^-PSCA-mAb	I^131^-PSCA-mAb inhibits cancer cell proliferation and enhances apoptosis in prostate cancer.	[139]
PSCA-CAR T cell	Phase I study shows effective primary antitumor effects on metastatic castration-resistant prostate cancer (mCRPC) at 100 million dose plus lymphodepletion (LD) chemotherapy.	[140]
PSA, PSCA, PSMA antigen	The high expression level of PSMA and PSCA is associated with an increase in the spread of prostate cancer, and it is possible to diagnose prostate cancer through peripheral blood with a combination of PSMA-PSA or PSCA-PSA antigens.	[141]
PSCA-CAR T cell	Final results from phase I PSCA-targeted 4-1BB-co-stimulated CAR T cell therapy indicate antitumor activity against mCRPC with dose-limiting toxicities (DLT) of cystitis and LD was required for T cell expansion and activity.	[142]
LU-Labeled PSCA antigen-specific monoclonal antibody	Due to the stable cell binding and specific accumulation in PSCA-positive tumor cells, radioimmnotherapy and imaging of prostate cancer is feasible using anti-PSCA monoclonal antibody.	[143]
UniCAR T cells	Due to its self-regulating capacity, UniCAR T cell can be selectively activaited by multiple target modules (TMs) such as binding to PSCA, which provoke release of pro-inflammatory cytokines, and PCa cell lysis both *in vitro* and *in vivo*.	[144]
Anti-PSCA CAR-T cells	Anti-PSCA CAR-T cells, specifically eliminated target tumor cells both in vivo and ex vivo and have the potential to treat patients with GC cancer in the clinic.	[145]
Engineered humanized anti-PSCA A2 scFv-Fc2 antibody with double mutation (A2DM, t1/2 12 h	Therapy with 177 Lu-A2DM in mice bearing KPC-hPSCA expressing pancreatic ductal adenocarcinoma (PDAC) showed marked reduction of tumor size to 105 ± 63 mm3 compared 1143 ± 754 mm3 for saline-treated at day 25.	[146]
Tandem (Tan) CAR-T targeting MUC1 and PSCA	Tan CAR T cell therapy has therapeutic effect in NSCLC and has a preclinical rationale for combination with anti-PD-1 antibody.	[147]
CAR-NK-92 cells	PSCA CAR-NK-92 cells show good anti-tumor effect on PSCA+ tumor cells both *in vitro* and *in vivo*, and has the potential to be a therapeutic strategy for cervical cancer.	[148]
α-RIT with ^211^At-labeled anti-PSCA A11 minibody	α-radioimmunotherapy (RIT) with astatine 211-labeled anti-PSCA A11 minibody, showed strong growth inhibition on both macrotumors and intratibial microtumors and promising treatment for micro metastatic and minimal residual disease of prostate cancer.	[149]
124I-A11 PSCA minibody	Phase I study in subjects with metastatic bladder, prostate and pancreatic cancer, 2013-2017	NCT02092948
PSCA-targeted CAR-T cells (BPX-601)	Phase I-II study on safety and activity of BPX therapy in patients with advanced solid tumors, 2016-2025	NCT02744287
PSCA-CAR T cells	Phase I study of PSCA CAR T cell therapy in PSCA+ metastatic castration-resistant prostate cancer, 2009-2023	NCT03873805

The in-situ hybridization method detects that PSCA is expressed in the epithelium of basal cells in normal prostate tissue. The epithelium of these cells contains progenitor cells that are required for the final differentiation of secretory cells using cytokeratin marker, it was found that there are at least two types of cellular subpopulation in the basal cell epithelium that PSCA is secreted by [44]. The PSCA antigen is expressed in most androgen-independent tumors and can also be detected in tumors that do not have an androgen receptor, and therefore could serve as a marker for androgen-independent tumors that have lost their functional androgen receptors [1]. In addition to differential PSCA expression, its polymorphisms are equally important in the development of numerous cancers, including diffuse and intestinal gastric cancer and can pave the way for the development of ethnic-based effective anti-cancer prognostics and therapies [72].

PSCA mRNA is overexpressed in prostate cancer and is associated with tumor stage, grade and androgen-independence which can be used for the diagnosis of prostate cancer. In addition, this relationship can be of adverse predictor for the recurrence and clinical progression or survival [46]. Importantly, the identification of cells that show overexpression of PSCA in bone marrow and peripheral blood of patients with prostate cancer may be a much better indicator for detecting and predicting metastatic progression than tests that examine only PSA-positive or PSMA-positive [6].

ISH and IHC methods have shown that PSCA mRNA expression is weak or not expressed in benign prostatic hyperplasia and low-grade PIN but is very high in HGPIN and may be involved in distinguishing between benign or cancerous prostate [150].

In addition to the prostate, PSCA antigen is expressed in some normal tissues and their cancer counterparts. Its expression pattern in breast cancer is, such that, PSCA protein in IMPC and IDC-NST acts as up-regulation and has the highest expression in these two types of breast cancer, while in ILC it acts as down-regulation and has the lowest expression, so targeted drug therapy for patients with overexpression of PSCA in IMPC may reduce the rate of cancer recurrence in these patients [70].

PSCA-based immunotherapeutic methods may be effective in treating patients who resist treatment to Her2/neu [54]. Interestingly, the expression of PSCA in normal and gastric cancer tissue is quite opposite, so in normal gastric tissue, the level of PSCA expression is higher than in cancerous tissue and may even be absent in cancerous tissue [13, 46]. PSCA function in this cancer, unlike prostate cancer, is down-regulation and acts as a tumor suppressor [55].

PSCA has a tumor-promoting role in brain tumors and given that PSCA is also expressed in normal neural and plexus cells of the brain, PSCA-based clinical applications may be limited due to possible side effects on the central nervous system [74]. In human lung cancer, high levels of PSCA are expressed, so that its inhibition dramatically inhibits the growth of cancer cells and therefore the PSCA antigen is a therapeutic target for this cancer. On the other hand, the targets of PSCA-based treatments in the treatment of prostate cancer, such as PSCA peptide vaccination against hormones and chemotherapy-refractory, which are in phase I/II clinical trials, can also be used for lung cancer to increase the survival rate of patients [59]. Due to the high expression of PSCA in NSCLC, CAR-PSCA T cell therapy showed that these engineered T cells can detect and kill PSCA-positive cancer cells and therefore CAR T cell therapy can be used as a new treatment for NSCLC patients [151]. PSCA in nasopharyngeal cancer NPC acts as a negative regulator for migration and invasion of these cells and modulates the metastatic process in a negative way in NPC cells by regulating the EMT program. In which the slug transcription factor plays a significant role, therefore, the participation of these two factors in EMT may design new therapies for the treatment of metastatic NPC patients and promise new hope for these patients [60]. In OSCC cancer, PSCA expression is high in cancerous tissue and may play a role in the development, prognosis and progression of this cancer, and its polymorphisms may also be involved in susceptibility to OSCC [116], but the molecular mechanism is far from clear. Thus, identifying the functional processes of this antigen in the OSCC may provide new insights into the study and treatment of this cancer. During the process of endometrial cancer (EAC), the presence of PSCA has also been identified and because in EAC, PSCA antigen is associated with age factor, it is considered as a marker for screening patients over 54 years. Also because of its association with tumor grade and metastasis, it is helpful in diagnosing tumor malignancy and can be a therapeutic target for EAC [119]. The function of PSCA in GBC or gallbladder cancer is down-regulation, and its overexpression in this cancer is associated with tumor differentiation, metastasis, tumor stage and survival time [106], so this may serve as a marker to detect tumor progression and patient survival rate, while PSCA haplotypes (T rs2294008–G-rs2978974) increases gallbladder cancer in women and therefore may be effective in screening processes.

PSCA overexpression in ESCC cancer is associated with some clinicopathological features such as poor differentiation, lymph node metastasis and advanced stage and is a tumor suppressor candidate in this cancer. Therefore PSCA down-regulation is considered as a prognostic predictor for ESCC patients [62]. In CC-RCC cancer, due to the high expression of PSCA in the advanced type of this cancer, the role of this marker in tumor invasion is clear and also due to its association with tumor differentiation, it plays an essential role in cancer progression [64]. PSCA can be exploited to assess the invasion and progression of tumors in this cancer and serve as a new marker in the diagnosis, treatment and prognosis of CC-RCC.

Finally, PSCA has a low level of expression in the normal bladder tissue and is expressed in the superficial layer of urothelium, but in cancerous tissue the level of expression increases. In normal and dysplastic tissue, is evident in the superficial layer of the urothelium, but in the CIS it is found in all neoplastic urothelium layers, therefore its expression may be used to differentiate between the CIS and other types of bladder cancer [65]. In this line, anti-PSCA mAb is injected intravenously to treat localized disease, especially CIS, or systemically for advanced bladder tumors [65]. In GTD cancer, PSCA overexpression is involved in the conversion of HM to GTN and its development, and can therefore be used as a new marker in predicting the progression of HM in addition to HCG assay [66].

## Conclusion

Together, PSCA is an original tumor biomarker for prostate cancer. However, its expression pattern as well as polymorphisms differ among different normal and cancer tissues and in different tumor stages. PSCA is a new biomarker and can be used as a prognostic marker for a variety of tumors as well as predicting survival rates in patients. PSCA monoallelic variants display clonal heterogeneity and there is no consistency in the expression level nor specific monoallelic variants with disease states in different populations. As for *H.pilori* infection, the C allele is a risk factor for gastric ulcers, while T variant promotes intestinal metaplasia. Likewise, for hormone-responsive tumors such as breast cancer, the age is not a predictive factor but for EAE (women > 54 y) and GC (age < 40 y) age is a risk factor. Collectively, in most cases, PSCA expression level is associated with disease state and survival in patients. More meta-analysis and DNA sequencing experiments are warranted to decipher the role of different PSCA variants and their relation with PSCA expression and cancer susceptibility in different populations. Nonetheless, PSCA is a valuable newly-emerged biomarker and therapeutic target for solid cancer and PSCA based CAR T cell therapy coupled with immunotherapy is now being actively pursued in clinical trials. In the future, theranostic devices can be developed for simultaneous imaging and targeted therapy of tumors, with high precision, cheaper and faster results over traditional methods such as IHC.

## Data Availability

Not applicable.

## Abbreviations

mAbs: Monoclonal antibodies
IHC: Immunohistochemistry
MALDI-TOF MS: Matrix-assisted laser desorption/ionization time-of-flight mass spectrometry
FISH: Fluorescence in situ hybridization
ORs: Odds ratios
CIs: Confidence intervals
PCR–RFLP: Polymerase chain reaction-restriction fragment length polymorphism
RT qPCR: Reverse transcription quantitative polymerase chain reaction
PDX: Patient-derived xenograft
EMT: Epithelial–mesenchymal transition
ELISA: Enzyme linked immunosorbent assay
TMAs: Tissue microarrays
PFS: Progression-free survival
TAT: Targeted alpha therapy
CAR: Chimeric antigen receptor
Tan CAR-T: Tandem CAR-T
TRT: Targeted radiopharmaceutical
PMFS: Poor metastasis-free survival
OS: Overall survival
DFS: Disease free survival
